# NOTCH1 Aberrations in Chronic Lymphocytic Leukemia

**DOI:** 10.3389/fonc.2018.00229

**Published:** 2018-06-27

**Authors:** Emanuela Rosati, Stefano Baldoni, Filomena De Falco, Beatrice Del Papa, Erica Dorillo, Chiara Rompietti, Elisa Albi, Franca Falzetti, Mauro Di Ianni, Paolo Sportoletti

**Affiliations:** ^1^Department of Experimental Medicine, Biosciences and Medical Embryology Section, University of Perugia, Perugia, Italy; ^2^Department of Life, Hematology Section, Health and Environmental Sciences, University of L’Aquila, Perugia, Italy; ^3^Institute of Hematology-Centro di Ricerche Emato-Oncologiche (CREO), University of Perugia, Perugia, Italy; ^4^Department of Medicine and Aging Sciences, University of Chieti Pescara, Chieti, Italy; ^5^Department of Hematology, Transfusion Medicine and Biotechnologies, Ospedale Civile, Pescara, Italy

**Keywords:** NOTCH1, chronic lymphocytic leukemia, prognostic biomarker, targeted therapy, gene mutation

## Abstract

Chronic lymphocytic leukemia (CLL) is an incurable B-cell neoplasm characterized by highly variable clinical outcomes. In recent years, genomic and molecular studies revealed a remarkable heterogeneity in CLL, which mirrored the clinical diversity of this disease. These studies profoundly enhanced our understanding of leukemia cell biology and led to the identification of new biomarkers with potential prognostic and therapeutic significance. Accumulating evidence indicates a key role of deregulated NOTCH1 signaling and *NOTCH1* mutations in CLL. This review highlights recent discoveries that improve our understanding of the pathophysiological NOTCH1 signaling in CLL and the clinical impact of *NOTCH1* mutations in retrospective and prospective trials. In addition, we discuss the rationale for a therapeutic strategy aiming at inhibiting NOTCH1 signaling in CLL, along with an overview on the currently available NOTCH1-directed approaches.

## Introduction

Chronic lymphocytic leukemia (CLL) is characterized by a clonal expansion of mature CD5+ CD23+ B-lymphocytes that accumulate in the bone marrow and infiltrate lymphoid tissues such as the spleen and lymph nodes (1). CLL, the most common leukemia in the Western world, is a heterogeneous disease and remains incurable in virtually all cases. CLL predominates in the elderly, and the incidence of the disease increases exponentially with age (2). Thus, the number of CLL patients is expected to rise in the future, given the increase in the aging population, bringing to light new clinical challenges and public health issues. Patients with CLL show a tremendously variable clinical course ranging from excellent prognosis with no treatment to short-term survival, despite early initiation of therapy (3). Genomic and molecular characterization of CLL has largely explained the heterogeneous clinical course of this disease, improving the prognostic risk stratification (4). Features predicting CLL outcome include somatic mutations of the immunoglobulin heavy chain variable (*IGHV*) genes, expression of CD38 and ZAP-70 surrogate markers, identification of chromosomal abnormalities (deletions of chromosome 13q, 17p, and 11q, and trisomy 12), and recurrent mutations in *TP53, NOTCH1*, and *SF3B1* genes (5).

*NOTCH1* has emerged as the most commonly mutated gene in CLL at diagnosis, and its frequency rises with disease progression (6–8). *NOTCH1* mutations are associated with poor outcomes and result in more difficulties to treat CLL (9–12). Mutated CLL shows a biologically active form of NOTCH1, though NOTCH1-dependent transcriptional responses have also been described in CLL cases lacking the mutation (13). Therefore, *NOTCH1* represents a new key cancer gene in CLL whose genetic and pathway alterations are likely to represent a novel oncogenic process in this disease.

In this review, we discuss the impact of NOTCH1 aberrations on the pathogenesis, prognosis, and therapeutic strategies in CLL, based on available literature.

## NOTCH1 Protein Structure and Pathway

**Figure 1:**
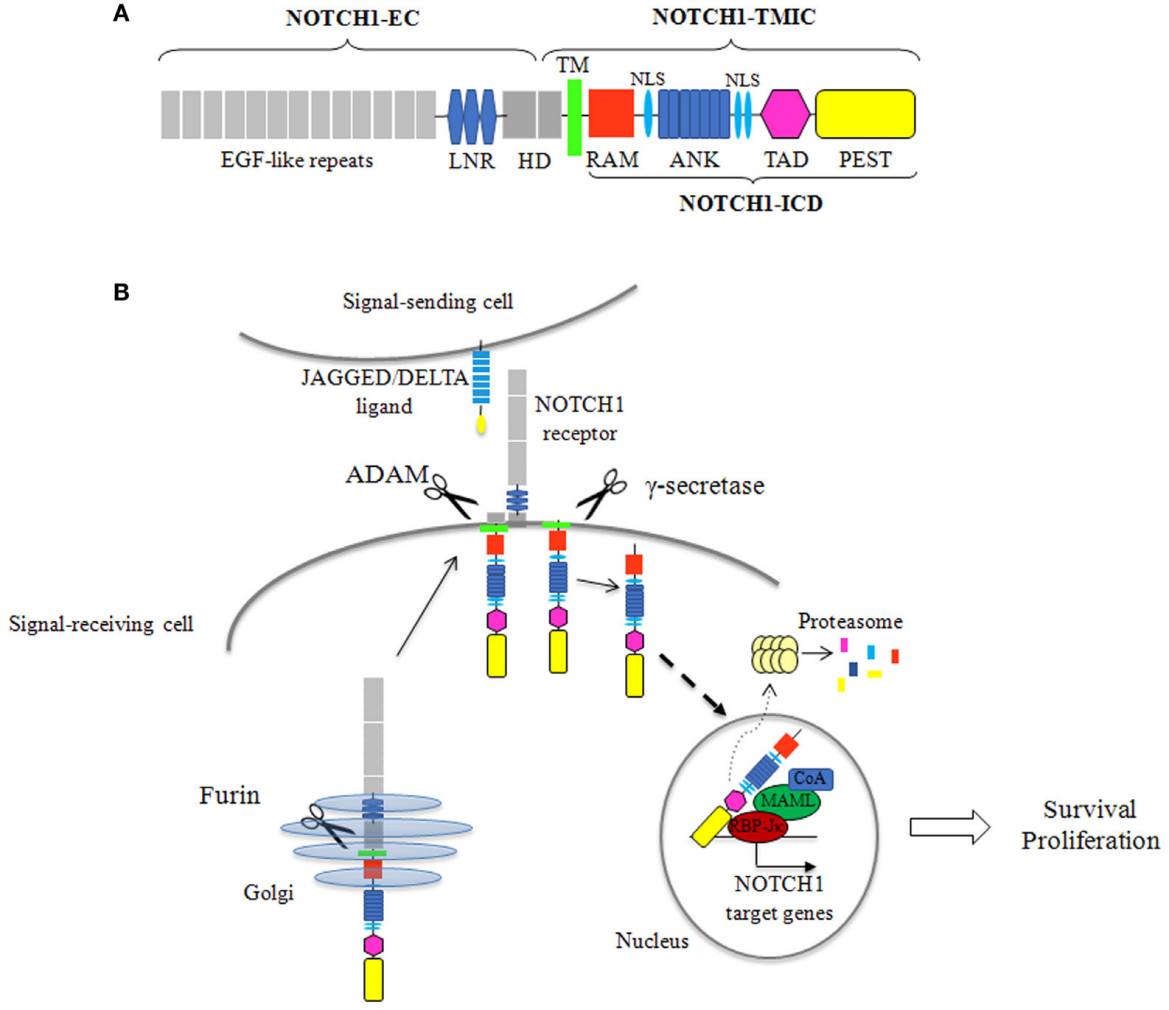
NOTCH1 protein structure and signaling activation. **(A)** The mature NOTCH1 receptor is a heterodimer composed of an extracellular subunit (NOTCH1-EC) and a transmembrane and intracellular subunit (NOTCH1-TMIC). The NOTCH1-EC includes epidermal growth factor (EGF)-like repeats, involved in ligand binding, three LIN-12/NOTCH repeats (LNR), which prevent receptor activation in the absence of ligands, and the heterodimerization domain (HD) involved in non-covalent interactions between the NOTCH1-EC and NOTCH1-TMIC. The NOTCH1-TMIC consists of the transmembrane domain (TM) and the intracellular domain (ICD) (NOTCH1-ICD). NOTCH1-ICD comprises an RBPJ-associated molecule (RAM) domain, seven ankyrin (ANK) repeats, nuclear localization signals (NLS), a transactivation domain (TAD), and a PEST domain, which is involved in proteasomal degradation of active NOTCH1-ICD. **(B)** Newly synthesized NOTCH1 precursor is cleaved by a furin-like convertase (Furin) in the Golgi apparatus to generate the mature receptor. NOTCH1 signaling initiates when a JAGGED or DELTA ligand expressed on a signal sending cell interacts with NOTCH1 on a signal receiving cell. This interaction triggers two sequential cleavages of NOTCH1: the first, by an a disintegrin and metalloproteinase (ADAM) metalloproteinase, generates the substrate for the second cleavage by γ-secretase, which releases the active NOTCH1-ICD. NOTCH1-ICD translocates to the nucleus where it forms a transcriptional activation complex by interacting with the transcription factor CSL/RBP-Jk, mastermind-like proteins, and others coactivators (CoA), leading to the expression of NOTCH1 target genes. In physiological conditions, NOTCH1 signal attenuation is mediated by ubiquitination and proteasomal degradation of NOTCH1-ICD.

NOTCH1 is a single pass transmembrane heterodimeric receptor. It is synthesized as a single precursor that undergoes a proteolytic cleavage by a furin-like convertase in the Golgi apparatus. The mature receptor expressed on the cell surface is composed of an N-terminal extracellular subunit (NOTCH1-EC) and a C-terminal transmembrane and intracellular subunit (NOTCH1-TMIC), held together by non-covalent interactions. The NOTCH1-EC contains a series of epidermal growth factor-like repeats, involved in ligand binding, and three LIN-12/NOTCH repeats that stabilize the heterodimerization domain (HD), preventing ligand-independent activation of the receptor. The NOTCH1-TMIC consists of a transmembrane region followed by different cytoplasmic domains that form the NOTCH1 intracellular domain (ICD) (NOTCH1-ICD). NOTCH1-ICD includes an RBPJ-associated molecule domain, a series of ankyrin (ANK) repeats, flanked by nuclear localization signals, a transactivation domain (TAD), and a C-terminal PEST domain, a region rich in proline (P), glutamic acid (E), serine (S), and threonine (T), which regulates stability and proteasomal degradation of active NOTCH1-ICD (14, 15) (Figure 1A). NOTCH1 signaling is triggered when a ligand, from the SERRATE/JAGGED or DELTA families, expressed on an adjacent cell, binds the receptor. This interaction starts two successive proteolytic cleavages: an extracellular juxtamembrane cleavage, by a disintegrin and metalloproteinase that occurs in the HD and generates the substrate for the intramembrane cleavage, by γ-secretase complex, resulting in the release of the active NOTCH1-ICD which translocates to the nucleus. In the nucleus, NOTCH1-ICD forms a transcription complex with the transcription factor RBP-Jk, mastermind-like (MAML) proteins and other coactivators, switching on the expression of NOTCH1 target genes (15). The signal is terminated through the ubiquitination of degron sites on the PEST domain, followed by proteasome-dependent degradation of the active NOTCH1-ICD (Figure 1B).

## History of NOTCH1 Signaling and Examination of Gene Alterations in CLL

Initially, NOTCH1 was considered essential to direct T-cell lineage commitment at the expense of B-cell development (16), and its oncogenic potential has been demonstrated in T-cell leukemia (17, 18). In a pioneering study, we demonstrated that CLL cells expressed high levels of NOTCH1 receptor together with its ligands JAGGED1 and JAGGED2. NOTCH1 was constitutively activated in CLL cells and contributed to their survival and resistance to apoptosis (19). Based on these observations, an initial *NOTCH1* gene defect in relation to CLL was reported in 2009, when our group identified a frameshift deletion of *NOTCH1* gene in a percentage of unselected CLL patients (6). Soon after, we determined the significant prognostic implication of *NOTCH1* mutation in a pivotal retrospective single-institution cohort study (9).

The advent of next-generation sequencing (NGS) technology confirmed the presence of stabilizing mutations of *NOTCH1* in several independent CLL groups (8, 20, 21). Fabbri et al. were the first to identify the link between *NOTCH1* mutations and chemotherapy refractoriness and disease progression to large cell lymphoma (7). Other publications further confirmed the impact of *NOTCH1* mutations on poor clinical outcome and delineated their association with other genetic markers (i.e., unmutated *IGHV* genes and trisomy 12) (22–24). Based on this observation, *NOTCH1* mutations have been integrated in the hierarchical cytogenetic prognostic stratification model devised by Döhner et al. that is still of major clinical relevance (5, 25). Nowadays, the recommendation for the assessment of *NOTCH1* mutations has not been introduced into general practice but is often performed in clinical trials. The analysis of prospective series yielded conflicting results with retrospective studies on the prognostic potential of NOTCH1 in CLL (12, 26). Genetic and molecular findings were paralleled by a number of biological studies aimed to define the leukemogenic role of *NOTCH1* mutations. Results of these studies will be discussed in detail in this review. More recently, the identification of non-mutational activation of NOTCH1 in CLL (13) implied that NOTCH1-ICD levels might represent a prognostic biomarker to refine the mutation/cytogenetic hierarchical model of risk stratification. Furthermore, these findings implicated a much broader role of NOTCH1 in CLL pathogenesis and raised the question about the mechanisms leading to the signaling activation.

## *NOTCH1* Mutational Status in CLL

### Types of *NOTCH1* Mutations in CLL

**Figure 2:**
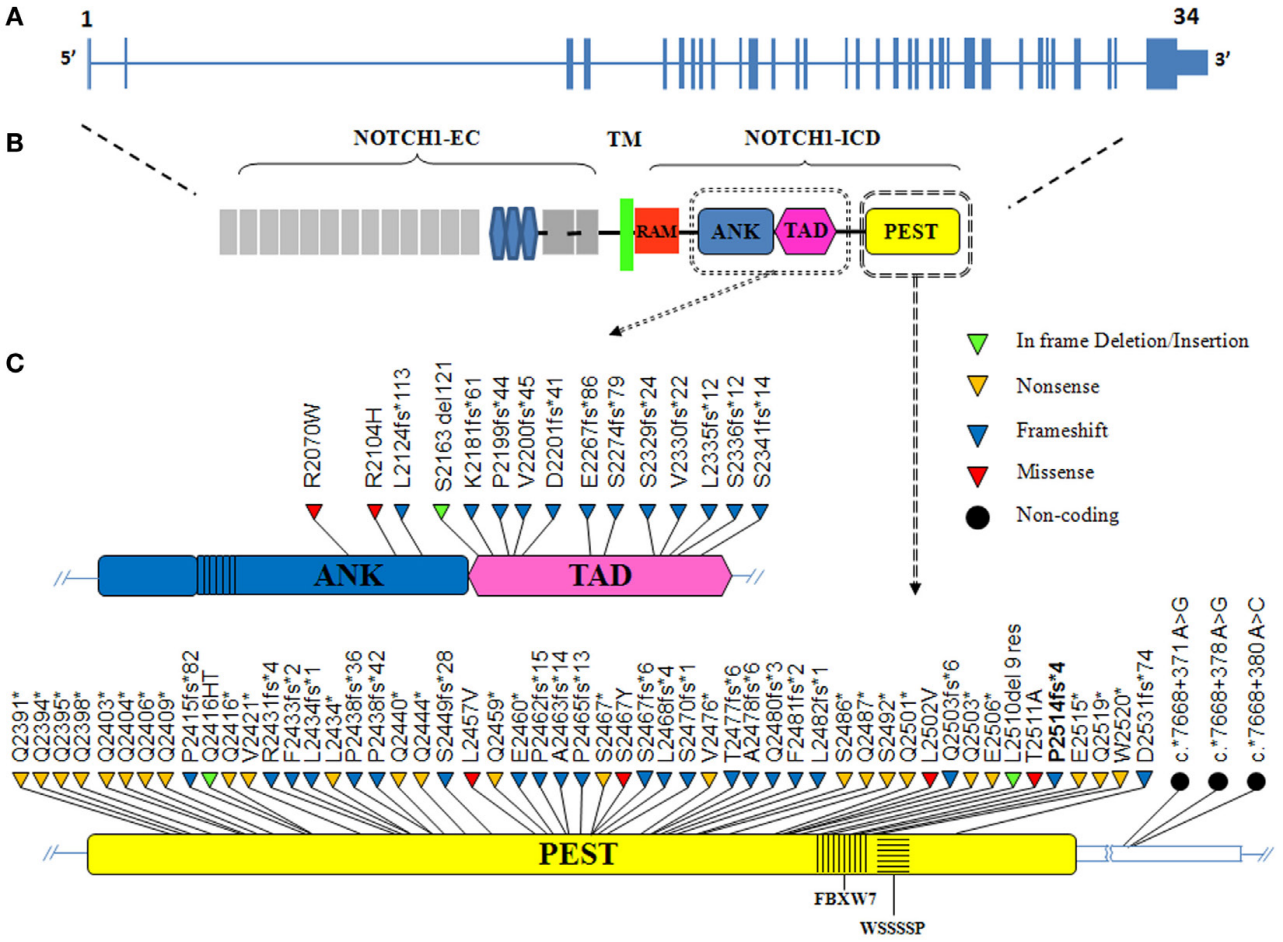
The distribution of *NOTCH1* mutations in chronic lymphocytic leukemia (CLL). **(A)** Schematic diagram of the human *NOTCH1* gene consisting of 34 exons (vertical bars) and spanning 50 kb. **(B)** The NOTCH1 protein includes an extracellular domain (EC) and an intracellular domain (ICD) linked by a transmembrane portion (TM). The NOTCH1-ICD consists of RBPJ-associated molecule (RAM) and ankyrin (ANK) domains involved in CSL/RBP-Jk binding, a transactivation domain (TAD) and a PEST (polypeptide enriched in proline, glutamate, serine and threonine) domain, which is a hotspot for mutation in CLL. **(C)** The ANK/TAD (upper panel) and PEST (lower panel) domains are magnified, and the color-coded shapes indicate the position of the in frame deletion/insertion (green), non-sense (yellow), frameshift (blue), missense (red), and non-coding (black dot) mutations found in CLL. The F-box and WD repeat domain-containing 7 (FBXW7) and WSSSSP degron are indicated within the PEST domain.

**Figure 3:**
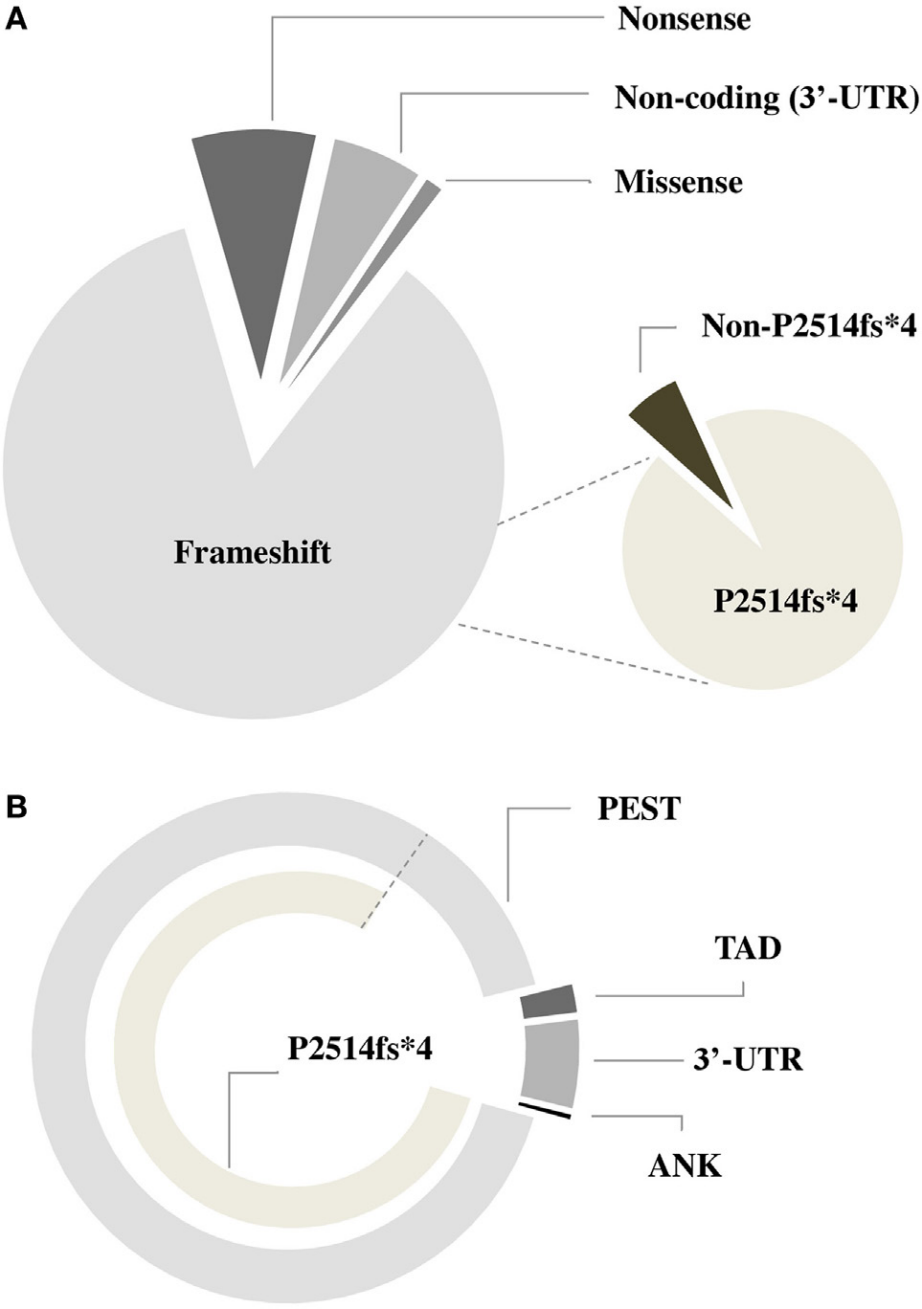
Frequency of different types of *NOTCH1* mutations in chronic lymphocytic leukemia. Pie charts describing the incidence of *NOTCH1* mutations according to the type of genetic variant **(A)** and domains **(B)** involved.

*NOTCH1* genetic alterations have been described in different human malignancies, including hematopoietic and solid tumors (27). Chromosomal rearrangements (28) and mutations (29) of the *NOTCH1* gene were initially described in T-cell acute lymphoblastic leukemia (T-ALL), which displayed aberrant activation of NOTCH1 signaling in over 60% of the cases. In T-ALL, mutations occur more frequently on the HD domain, resulting in the activation of the receptor independent of ligand binding (29, 30). Unlike T-ALL, the most common *NOTCH1* mutation in CLL affects the C-terminal PEST domain causing prolonged half-life of the cleaved protein (Figure 2). This mutation, accounting for approximately 80% of cases (Table 1; Figure 3) consists in a 2-bp (CT) frameshift deletion (c.7541_7542delCT) localized in the exon 34 that generates a premature stop codon causing truncations of the C-terminal PEST domain (P2514fs*4) (6). The truncated protein lacks the F-box and WD repeat domain-containing 7 (FBXW7) and WSSSSP degron domains required to target activated NOTCH1 for proteasomal degradation (Table S1 in Supplementary Material). This leads to increased NOTCH1-ICD stability and aberrantly prolongs its activation, indicating that mutations may contribute to enhance NOTCH1 signaling in CLL. The reasons for which *NOTCH1* mutations affect two different domains in CLL and T-ALL samples remain unclear. We can hypothesize the existence of distinctive genetic contexts that probably depend on highly conserved mechanisms. Alternatively, the domain targeted by mutation may depend on either a distinct epigenetic regulation in T versus B cells or on a selective pressure from microenvironmental conditions.

**Table 1:** Frequency of genetic variants of *NOTCH1* mutations across different chronic lymphocytic leukemia studies.

No. patients	Mutation	Domain	%
646	P2514fs*4	PEST	78.78
35	c.*7668+371 A>G	UTR	4.2
14	Q2444*	PEST	1.7
10	L2482fs*1	PEST	1.2
10	c.*7668+378 A>G	UTR	1.2
6	Q2503*	PEST	0.73
5	Q2394*	PEST	0.61
4	Q2440*	PEST	0.48
4	Q2501*	PEST	0.48
3	S2336fs*12	TAD	0.36
3	S2341fs*14	TAD	0.36
3	Q2404*	PEST	0.36
3	A2463fs*14	PEST	0.36
3	F2481fs*2	PEST	0.36
3	Q2519*	PEST	0.36
2	Q2403*	PEST	0.24
2	Q2406*	PEST	0.24
2	Q2409*	PEST	0.24
2	P2415fs*82	PEST	0.24
2	Q2416HT	PEST	0.24
2	Q2416*	PEST	0.24
2	V2421*	PEST	0.24
2	Q2459*	PEST	0.24
2	S2470fs*1	PEST	0.24
2	V2476*	PEST	0.24
2	A2478fs*6	PEST	0.24
2	c.*7668+380 A>C	UTR	0.24
1	R2070W	ANK	0.12
1	R2104H	ANK	0.12
1	L2124fs*113	ANK	0.12
1	S2163del121	TAD	0.12
1	K2181fs*61	TAD	0.12
1	P2199fs*44	TAD	0.12
1	V2200fs*45	TAD	0.12
1	D2201fs*41	TAD	0.12
1	E2267fs*86	TAD	0.12
1	S2274fs*79	TAD	0.12
1	S2329fs*24	TAD	0.12
1	V2330fs*22	TAD	0.12
1	L2335fs*12	TAD	0.12
1	Q2391*	PEST	0.12
1	Q2395*	PEST	0.12
1	Q2398*	PEST	0.12
1	R2431 fs*4	PEST	0.12
1	F2433fs*2	PEST	0.12
1	L2434fs*1	PEST	0.12
1	L2434*	PEST	0.12
1	P2438fs*36	PEST	0.12
1	P2438fs*42	PEST	0.12
1	S2449fs*28	PEST	0.12
1	L2457V	PEST	0.12
1	E2460*	PEST	0.12
1	P2462fs*15	PEST	0.12
1	P2465fs*13	PEST	0.12
1	S2467*	PEST	0.12
1	S2467Y	PEST	0.12
1	S2467fs*6	PEST	0.12
1	L2468fs*4	PEST	0.12
1	T2477fs*6	PEST	0.12
1	Q2480fs*3	PEST	0.12
1	S2486*	PEST	0.12
1	Q2487*	PEST	0.12
1	S2492*	PEST	0.12
1	L2502V	PEST	0.12
1	Q2503fs*6	PEST	0.12
1	E2506*	PEST	0.12
1	L2510 del9 res	PEST	0.12
1	T2511A	PEST	0.12
1	E2515*	PEST	0.12
1	W2520*	PEST	0.12
1	D2531fs*74	PEST	0.12

NGS studies in CLL revealed several mutations other than the hotspot dinucleotide deletion targeting the function of the C-terminal PEST domain (8, 21, 31–34) (Table 1; Figure 3). These genetic alterations include frameshift mutations or truncations that affect different nucleotides in exon 34 and occur with lower frequency than that of the canonical delCT mutation (12.5%). The majority of these mutations lead to the disruption of the Cdc phosphodegron domain targeted by FBXW7 and the following WSSSSP sequence. Rare non-delCT mutations include Q2519* and W2520* substitutions (35, 36) that result in the removal of the WSSSSP sequence alone. Similar alterations have been shown to be leukemogenic in T-ALL, suggesting a specific function in CLL. In addition, PEST domain mutants include a small number of missense mutations (34), some of which are reported in the single-nucleotide polymorphism database, suggesting their limited role in CLL. The TAD and ANK domains are targets of frameshift non-sense mutations in 1.9 and 0.4% of CLL, and generally determine a premature stop codon.

In 2015, Puente et al. reported recurrent mutations in the 3′ untranslated region (3′-UTR) of the *NOTCH1* gene of previously untreated CLL or monoclonal B-cell lymphocytosis (MBL) cases (32). The presence of these mutations was confirmed at low frequency (2–4%) in heterogeneous CLL cohorts of retrospective studies and clinical trials (33, 37). The most common target of 3′-UTR mutations is the non-coding region of the exon 34 at position 7668+371A>G, 7668+378A>G, and less frequently, 7668+380A>C (74.5, 21.3, and 4.2%, respectively). Each of these non-coding variants creates a new splice acceptor site that favors an alternative splicing event with a cryptic donor site located in the coding exon 34. As a result, most of the coding bases of the PEST sequence are removed, resulting in an increased NOTCH1 protein stability as in the case of delCT mutation. In most cases, 3′-UTR mutations are mutually exclusive with other *NOTCH1* somatic variants, supporting the analysis of exon 34 non-coding region to identify additional patients with pathogenic *NOTCH1* mutations. Preliminary evidence showed that patients with different *NOTCH1* mutations display constitutive levels of cleaved protein whose size is consistent with the type of mutations (34). This observation suggests potential differences in biological and prognostic impacts on CLL that need to be further analyzed. Besides mutations in the *NOTCH1* gene itself, several NOTCH1 pathway regulatory genes such as *FBXW7*, mediator complex subunit 12 (*MED12*), and spen family transcriptional repressor (*SPEN*) were also identified as mutated with low frequency in CLL. *FBXW7* and *MED12* loss-of-function mutations preventing proteasomal degradation of NOTCH1 were present in 2–5% CLL (20, 38). SPEN is a co-repressor of RBPJ and a putative negative regulator of NOTCH1 signaling. Inactivating mutations of the *SPEN* gene were detected in approximately 1% of CLL cases (39).

In addition to NOTCH1, CLL cells also express the NOTCH2 receptor which is constitutively activated (19). Despite NOTCH2 signaling appears to have a role in CLL cell survival similar to that of NOTCH1 (19, 40, 41), *NOTCH2* mutations have not been detected in CLL (42, 43). By contrast, *NOTCH2* mutations have been found in other non-Hodgkin B-cell lymphoma subtypes, such as splenic marginal zone lymphoma (SMZL) and diffuse large B-cell lymphoma (DLBCL). In DLBCL, *NOTCH2* mutations affect approximately 8% of patients with some cases having increased copies of the mutated *NOTCH2* allele (44). In SMZL, *NOTCH2* mutations represent the most recurrent genetic lesion accounting for approximately 20–25% of cases (42, 45, 46). Most of identified mutations were frameshift or non-sense mutations affecting PEST domain and resulting in protein truncation and increased NOTCH2 activation (45). Remarkably, NOTCH2 signaling has been shown to play a key role in marginal zone B-cell development in the spleen, and to be dispensable for the development of other B-cell lineages (47, 48).

Based on these observations, one can hypothesize that the selective requirement of NOTCH2 signals for the development of normal splenic marginal zone B cells provides a functional basis for the involvement of *NOTCH2* mutations in SMZL and not in other B malignancies, such as CLL, that derive from other cell types. In agreement with this hypothesis, the selective pressure to acquire *NOTCH1* mutations in CLL presumably reflects a special context-dependent role for NOTCH1 activation in normal naïve and memory B cells (13), which are considered the cells of origin of CLL (49, 50). The involvement of NOTCH1 alterations in CLL leukemogenesis will be detailed in a specific section of this review.

### Frequency of *NOTCH1* Mutations in CLL

**Figure 4:**
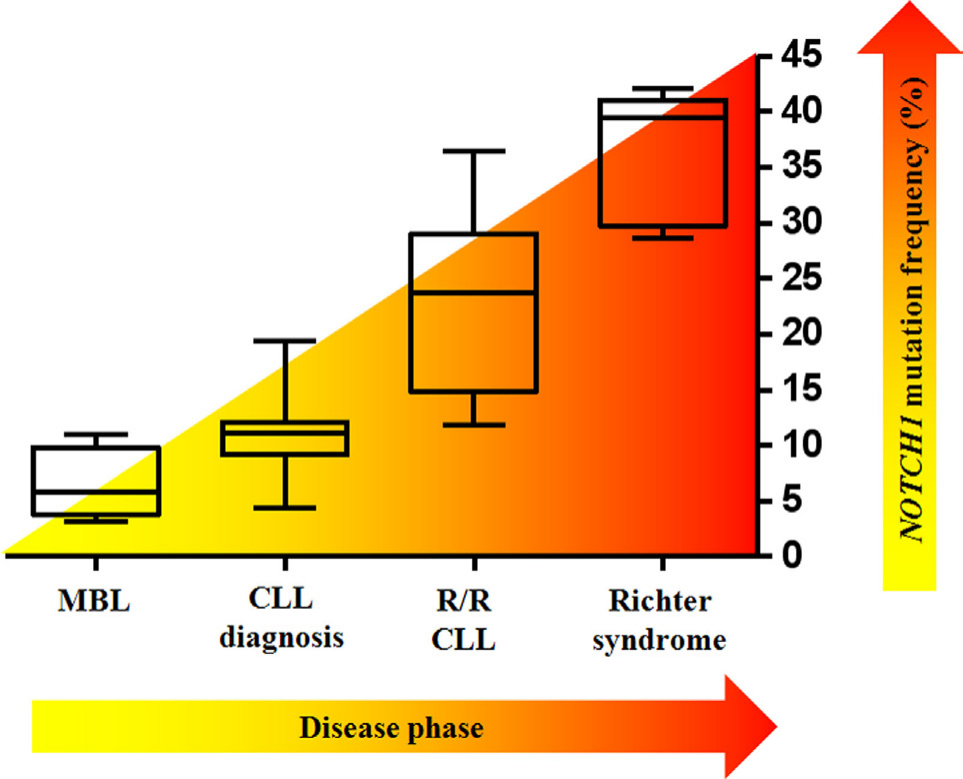
*NOTCH1* mutation frequency varies according to the clinical time point of the disease. Box–whisker plots indicate the prevalence of mutated NOTCH1 in monoclonal B-cell lymphocytosis (MBL), in chronic lymphocytic leukemia (CLL) at diagnosis, in relapsed/refractory (R/R) CLL, and in Richter syndrome.

**Figure 5:**
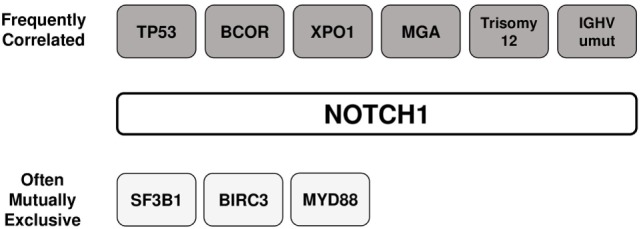
Association between *NOTCH1* mutations and other genetic features in chronic lymphocytic leukemia. The patterns of co-occurrence and mutual exclusivity between *NOTCH1* mutations and other genetic features are shown.

The prevalence of *NOTCH1* mutations in CLL varies greatly across studies, depending on differences between cohorts relative to time from diagnosis, stage of the diseases analyzed (Figure 4), and other genetic alterations enriched in the study. The frequency of mutated cases is between 6 and 12% at initial diagnosis of CLL, whereas only approximately 3% of patients with MBL harbor a mutation (51, 52). The average mutation rate increased to approximately 15–20% when considering only patients with fludarabine-refractory CLL (7). *NOTCH1* lesions are much more prevalent after disease progression to Richter transformation relative to newly diagnosed CLL, with 30% patients harboring mutations, mostly the classical delCT (7, 53). A high mutation rate has been described in independent cohorts of CLL cases with trisomy 12 (23, 54). Specifically, mutations were predominant in CLL with isolated trisomy 12, and less common in cases associated with additional chromosomal aberrations (55). Balatti et al. reported a mutation frequency of 41.9% in aggressive trisomy 12 cases, suggesting a critical role of NOTCH1 activation in this CLL subgroup (22). In addition, *NOTCH1* mutations frequently occur with deletions of the long arm of chromosome 14 (56), and are inversely correlated with chromosome 11 deletions (23). *NOTCH1* lesions are considerably associated with flow cytometry-based markers of poor prognosis including ZAP-70, CD38, and CD49d (26, 57–59). Analyses on larger number of patients documented a high frequency of unmutated *IGHV* genes in both coding and non-coding *NOTCH1*-mutated CLL cases (60). Evidence indicates that *NOTCH1* mutations can occur in the context of other genetic variables. The concurrent presence of *NOTCH1* and *TP53* mutations has been described in 1.2–2.6% of CLL patients (12, 21), and single cell analysis revealed that gene defects preferentially affected the same leukemic cells (61). Additional studies observed a high co-occurrence of mutations in *MGA, BCOR* (32), and *XPO1* genes (62) with those in *NOTCH1*. Interestingly, *NOTCH1* mutations were found to be mutually exclusive with *SF3B1, BIRC3*, and *MYD88* mutations (63). The patterns of co-occurrence and mutual exclusivity between *NOTCH1* mutations and other genetic features in CLL are shown in Figure 5.

The molecular method used to identify *NOTCH1* mutations represents one important factor that influences observed mutation rates (Table 2). Earlier studies used standard Sanger sequencing to detect delCT variants in the exon 34 region, therefore mutations with allelic frequencies below 20% were not detected (6, 9, 64). Similarly, early NGS techniques discarded variants below the sensitivity of Sanger sequencing, allowing the identification of clonal mutation in 4–12% CLL patients (7, 8). In many subsequent NGS-based analyses, deep sequencing allowed to sequence a genomic region even thousands of times and to detect rare subclonal mutations (≤1%). This type of approach permitted to reveal a mutation frequency of the *NOTCH1* gene between 12 and 25.5% (33, 34, 65). The presence of recurrent delCT mutations was investigated by high-sensitivity polymerase chain reaction (PCR)-based methods (i.e., allele-specific and droplet digital PCR) to identify the *NOTCH1* mutant in up to half of CLL patients (66–68). These techniques help uncover subclonal mutations and may be used to identify patients in need of close clinical follow-up or for prospective minimal residual disease studies.

**Table 2:** Comparison between different molecular methods used to identify *NOTCH1* mutations.

Approach	Target region	Limit of detection (%)	Frequency (%)		Reference
				
			Clonal	Subclonal included	
Sanger	Exons 26, 27, and 34	10	4.6–12.1	N.A.	Di Ianni et al. (6), Quesada et al. (31), and Villamor et al. (58)
WGS/WES	All genome and exome	10	8.3–12.2	N.A.	Puente et al. (8) and Fabbri et al. (7)
ARMS-PCR	7541_7542delCT	10	11.0	N.A.	Rossi et al. (10)
Pyrosequencing	Exons 25, 28, and 34	2.5	13.0	17.0	Kluk et al. (69)
NGS (454)	Exons 33 and 34	2	10.5	13.2	Weissmann et al. (21) and Jeromin et al. (36)
AS-PCR	7541_7542delCT	0.1	10.2	20.1	Sportoletti et al. (66)
NGS ultra-deep targeted	Exons 26, 27, and 34 and 3′-UTR	0.3–1	11.8–14.3	18.6–25.5	Nadeu et al. (33), Pozzo et al. (70), and D’Agaro et al. (34)
ddPCR	7541_7542delCT	0.03	18.1	53.4	Minervini et al. (68)
Fragment Analysis	7541_7542delCT	5	8.4	8.4	Campregher et al. (67)
Real-time PCR	7541_7542delCT	10	10.1	N.A.	Bilous et al. (71)
HRM	7541_7542delCT	1	6.02	6.02	Xu et al. (72)

*WGS, whole-genome sequencing; WES, whole-exome sequencing; ARMS-PCR, amplification refractory mutation system PCR; NGS, next-generation sequencing; NGS (454), next-generation sequencing (Roche 454 sequencing system); AS-PCR, allele-specific PCR; ddPCR, droplet digital PCR; HRM, high-resolution melting; N.A., not applicable*.

## Prognostic and Predictive Impact of *NOTCH1* Mutations in CLL

*NOTCH1* mutations have been strongly associated with clinical outcomes, making them ideal prognostic biomarkers for an accurate risk-adapted stratification of CLL patients. The following paragraphs provide a comprehensive review of the most relevant findings on the role of *NOTCH1* in CLL prognosis.

### *NOTCH1* Mutations in Retrospective CLL Studies

Several retrospective analyses demonstrated that patients harboring *NOTCH1* mutations have altered survival statistics and treatment outcomes compared with patients with the wild-type *NOTCH1*. In 2010, we first demonstrated that the presence of a 2-bp frameshift deletion (c.7541_7542delCT) localized in the exon 34 of *NOTCH1* reduced time from diagnosis to initial treatment in a small cohort of 133 CLL patients (9). Thereafter, two unbiased whole-exome studies of large European CLL cohorts also identified *NOTCH1* mutations in different functional domains (7, 8) associated with a significantly shorter overall survival (OS). Additional analysis of numerous independent cohorts of patients confirmed the adverse clinical outcome of *NOTCH1*-mutated CLL in univariate analysis (21, 36, 43, 58, 66, 73–75). Reasons for being allocated to a high-risk category could be explained by a strong correlation of *NOTCH1* mutations with other markers of poor prognosis, including unmutated *IGHV*, high ZAP-70 expression, and trisomy 12. Conflicting data about the independent prognostic effect of the *NOTCH1* mutant in CLL have been reported. According to Rossi et al., multivariate analysis identified *NOTCH1* mutation as an independent predictor of shorter survival, similar to that observed with *TP53* abnormalities (10). By contrast, *NOTCH1* mutations did not retain independent significance as a predictor of time-to-first treatment in one of the largest series of general practice CLL patients (76).

### *NOTCH1* Mutations in Clinical Prospective CLL Trials

The role of *NOTCH1* has been further refined shifting from the investigation of heterogeneous cohorts of retrospective studies to clinical trials with well-characterized CLL patients. The LRF CLL4 study was the first to validate the importance of *NOTCH1* gene mutations in the context of a prospective trial. In this context, *NOTCH1* was a prognostic biomarker of OS identifying patients with intermediate survival rather than the poor survival associated with *TP53*-deleted or -mutated individuals (26). Conversely, *NOTCH1* was not confirmed to be an independent prognostic factor for progression-free survival (PFS) in both the CLL4 and CLL8 studies (12, 26), employing either less intensive therapies or fludarabine-based CLL treatments. Similar information emerged from the analysis of the Gruppo Italiano Malattie Ematologiche dell’Adulto LLC0405 protocol that used the association of fludarabine and alemtuzumab in high-risk CLL (57), while *NOTCH1* mutation was unexpectedly identified as an independent favorable marker for PFS in fludarabine-refractory patients treated with alemtuzumab in the CLL2H trial of the German CLL Study Group (77). Allogeneic stem cell transplantation (Allo-SCT) offers the only potentially curative treatment option for patients with poor-risk disease. In the CLL3X trial, the use of reduced-intensity Allo-SCT provided long-term disease control in CLL patients independent of the presence of different adverse prognosticators including *NOTCH1, SF3B1*, and *TP53* mutations (78).

### *NOTCH1* Mutations and New Targeted CLL Treatments

Besides NOTCH1, several other signaling pathways and molecules, including the B-cell receptor (BCR)-associated kinases, Bruton tyrosine kinase (BTK) and PI3K, and the antiapoptotic protein BCL-2, are altered in CLL contributing to disease pathogenesis and representing new therapeutic targets. Indeed, the advent of agents inhibiting these key CLL players has dramatically changed treatment algorithms of high-risk CLL and downscaled the role of Allo-SCT as the most effective treatment for this disease. Clinically relevant CLL targeted drugs include inhibitors of the BTK (Ibrutinib) and PI3Kδ (Idelalisib) pathways and the antagonist of the antiapoptotic protein BCL-2 (Venetoclax). The results of randomized clinical trials demonstrated impressive activity of Ibrutinib as single agent for the treatment of relapsed/refractory disease (79) and del 17p CLL patients (80). The drug is well tolerated in the vast majority of patients although there are some common side effects, including an increased rate of clinically significant bleeding and atrial fibrillation, that have to be managed to optimize outcome (81). Its efficacy in relapsed patients as well as its tolerability has led to its increased use in previously untreated patients, especially in those with poor prognostic markers and/or the elderly. A recent report on a 5*-*year experience showed that Ibrutinib is associated with a high overall response rate of 89% with complete response rates increasing over time to 29% in treatment-naïve patients and 10% in relapsed/refractory patients (82).

Based on a randomized clinical trial, Idelalisib in combination with rituximab appeared to benefit pre-treated patients with CLL and showed equivalent activity in patients with and without abnormalities of the TP53 pathway (83). Recent evidence demonstrated the lack of adverse prognostic impact of complex karyotype on Idelalisib-treated patients (84) Despite efficacy of Idelalisib in CLL, adverse effects are common and often limit treatment.

Venetoclax is the first BCL-2 inhibitor to enter routine clinical practice. In a phase I study, Venetoclax induced durable responses in 79% of patients with relapsed/refractory CLL, including complete remissions in 20% of patients (85). The antileukemic effects of Venetoclax occurred rapidly, with high response rates, independent of negative prognostic indices. Reductions in tumor burden were consistent and deep in most patients, with the depth of response increasing over time. In the setting of clinical trials, Venetoclax is undergoing testing for use in treatment-naive patients and in combination with other new agents.

The marked benefit of CLL drugs targeting the BCR signaling or BCL-2 has been investigated in the contest of NOTCH1 alterations. In particular, the presence of a *NOTCH1* mutation did not negatively affect the efficacy of Ibrutinib on disease progression outcomes in the extended follow-up from the RESONATE study of relapsed/refractory CLL (86). On the other hand, Idelalisib showed a shorter duration of response in *NOTCH1*-mutated CLL compared with unmutated patients in a phase I trial (87). Recently, the association of *NOTCH1* mutation and low BAX/BCL-2 ratio showed synergistic prognostic properties in patients treated with Ibrutinib, identifying a CLL subset with reduced OS and PFS (88). These data support the rationale to improve the efficacy of Ibrutinib in *NOTCH1*-mutated CLL by using the BCL-2 inhibitor Venetoclax.

In addition, innovative CLL therapies include novel immunotherapeutic regimens using new anti-CD20 antibodies, immune checkpoint inhibitors, and adoptive immunotherapy using modified T lymphocytes (89). To date, only intravenous obinutuzumab, a novel antibody that targets CD20, in combination with chlorambucil, has entered clinical practice as a first-line treatment for patients with CLL (90). The predictive role of *NOTCH1* mutation in the context of immunotherapy will be discussed in a specific section of this review.

### Role of *NOTCH1* Mutations in New Integrated CLL Scoring Systems

Collectively, published data support mild negative effects of *NOTCH1* mutations in the prognosis of CLL, and such effects have been incorporated into novel prognostic scoring systems. Rossi et al. combined the genetic status of *NOTCH1* and other CLL mutations to the cytogenetic profile (5). The accuracy of survival prediction is significantly improved by including *NOTCH1* in the cytogenetic hierarchical model (25), leading to reclassification of approximately 20% of low-risk patients into higher-risk classes. According to this integrated model, *NOTCH1*-mutated patients belong to an intermediate-risk group that accounts for approximately 15–20% of newly diagnosed CLL and shows a 10-year survival of 37%. In particular, the detection of clonal *NOTCH1* mutations has allowed to refine the conventional fluorescence *in situ* hybridization-based prognostic stratification of trisomy 12 CLL patients (23, 91).

### Clinical Impact of Subclonal *NOTCH1* Mutations in CLL

Clinical information on *NOTCH1* mutations is mainly restricted to lesions represented in more than 10% leukemic cells, the limit of detection for Sanger sequencing. The high sensitivity of PCR methods and ultra-deep NGS allowed the accurate detection of low allele frequency somatic mutations in CLL. Recently, the presence of few *TP53* clones has been associated with poor CLL outcome (92). However, the prognostic impact of *NOTCH1*-mutated subclones is still controversial. We demonstrated that the presence of subclonal *NOTCH1* mutations from early phases of the disease affected patient survival, providing a proof-of-principle that very few leukemia subclones detected at diagnosis are important drivers of the subsequent disease course (66). Ultra-deep NGS revealed subclonal *NOTCH1* mutations that predicted shorter time-to-first treatment irrespective of *IGHV* mutational status (33). Conversely, this approach failed to find statistically significant differences in OS between patients harboring small subclonal mutations of *NOTCH1* and wild-type patients in two independent cohorts with similar numbers of patients (33, 93). These data have been confirmed in a larger cohort of 1,000 patients, where the presence of subclonal *NOTCH1* mutations influenced time to the first treatment but not OS of CLL patients (34). These data suggest the need for additional studies with large uniformly treated datasets to determine whether an allele frequency cutoff is necessary when evaluating these mutations in relation to clinical outcome.

### Clinical Impact of Non-delCT *NOTCH1* Mutations and Concurrent Genetic Abnormalities

Screening of *NOTCH1* mutations in CLL identified several genetic alterations outside the canonical region involved in delCT, but little is known about the association between different types of *NOTCH1* mutations and clinical outcome. These mutations are localized both in the coding sequence and 3′-UTR of exon 34, resulting in impaired protein degradation (32). Regardless of the mutation type, all *NOTCH1*-mutated cases retain their adverse prognostic impact on time-to-first treatment in a large retrospective analysis (34). In univariate analysis, patients with 3′-UTR mutations behaved similarly to patients with coding mutations in *NOTCH1* in terms of OS (32). Conversely, the independent impact of non-coding mutations on OS was either non-significant or not reliably assessed because of the small number of cases (60). The distinctive prognostic impacts of different *NOTCH1* mutations can be attributed to their capacity to generate NOTCH1-cleaved proteins with different sizes, suggesting unique leukemogenic effects that need further investigation. One hypothesis is that the presence of a mutated NOTCH1 protein with a specific configuration may correlate with a more sustained NOTCH1 signaling deregulation. As outlined elsewhere in this review, mutations in *NOTCH1* primarily result in premature protein truncation that prevents protein degradation. In this respect, different frameshift mutations may lead to the deletion of distinct portions of NOTCH1 degrons, with a potential impact on the inactivating phosphorylation of the protein. We might not exclude that mutated NOTCH1 proteins acquire additional functions contributing to stabilize the constitutive activation of the signaling.

### *NOTCH1* Mutation as a Predictive Factor

Recent reports provided evidence of an association between *NOTCH1* mutations and lack of benefit of CD20 antibody therapies, suggesting that NOTCH1 could have predictive potential. In the CLL8 trial, patients carrying *NOTCH1* mutations did not benefit of the inclusion of rituximab to standard fludarabine and cyclophosphamide (FC) chemotherapy, displaying a significantly lower PFS than that of *NOTCH1* wild-type patients and comparable to those treated with the FC protocol (12). These data were further confirmed by the analysis of homogeneously prospective CLL series treated with chemoimmunotherapy followed by a rituximab-based consolidation (94). Ofatumumab and obinutuzumab represent new generations of anti-CD20 monoclonal antibodies that have been developed for potential benefits over rituximab. *NOTCH1*-mutated patients randomized to ofatumumab in the RESONATE trial fared significantly worse than their non-mutated counterparts (86). In addition, *NOTCH1* gene mutation appears to predict reduced efficacy of ofatumumab in relapsed/refractory CLL, according to data from the phase III COMPLEMENT 2 trial. Conversely, mutational status of *NOTCH1* gene did not affect the B-cell depletion efficacy of obinutuzumab against CLL *ex vivo*. These data suggest a potential improved clinical outcome, although direct comparison between *in vitro* and *in vivo* data should be considered with caution. More recently, Estenfelder et al. described the predictive power of the *NOTCH1* gene mutation in 689 patients enrolled in the CLL11 protocol. In this study, obinutuzumab in addition to chlorambucil improved PFS compared with what was observed in patients treated with rituximab and chlorambucil in the subgroups with mutated *NOTCH1* (95). The application of all these findings to clinical practice is not yet fully defined, as these data need confirmation in independent cohorts before being applied in routine practice.

## Role of NOTCH1 Alterations in CLL Leukemogenesis

### NOTCH1 Alterations in the Transformation Process of Mature B Cells Into CLL Cells

Chronic lymphocytic leukemia has traditionally been considered a malignancy originating from oncogenic transformation of a mature antigen-experienced B cell (96) with a gene expression profiling related to normal CD27+ memory B cells (50, 97). The discovery that CLL cells could be distinguished by the presence or absence of *IGHV* gene mutations led to postulate that antigenic stimulation of naïve B cells might proceed either through a T cell-dependent reaction occurring in the germinal center (GC) and leading to CLL with mutated *IGHV* genes, or in a T cell- and GC-independent manner leading to CLL with unmutated *IGHV* genes (98). Recent studies revealed that *IGHV*-mutated CLL was derived from a previously unrecognized CD5+ CD27+ post-GC memory B cell subset (49), whereas *IGHV*-unmutated CLL was generated from pre-GC CD5+ CD27− B cells that were derived from naïve B cells, a separate lineage of B cell precursor or GC-independent memory B cells (4).

A growing body of evidence indicated that a major driver of CLL pathogenesis was the BCR signaling (99–101), which provided a survival and proliferative advantage to CLL cells, leading to malignant clone selection (98). Given that CLL cells often carry stereotyped BCR, it is likely that a role in the leukemic clone selection is played by recognition of common epitopes or classes of structurally similar epitopes of autoantigens or microbial antigens (102–104). Even normal neighboring cells within the proliferation centers of peripheral lymphoid tissues may favor cell growth or prevent apoptosis of the leukemic clone by providing essential cell–cell and cell–soluble factor interactions (105, 106). Interestingly, it has been shown that stromal cells within lymph nodes expressed the NOTCH1 ligand JAGGED1 that induced NOTCH1 activation in CLL cells (107), suggesting that an aberrant NOTCH1 activity might contribute to CLL development. In line with this hypothesis, the evidence that NOTCH1 is activated in normal naïve, and memory B cells together with the finding that NOTCH1 regulates genes involved in normal B-cell physiology, suggests that in CLL, NOTCH1 activation is the consequence of a dysregulated physiologic signal (13). The genes upregulated by NOTCH1 transcriptional activity were mainly involved in the survival and proliferation of mature B cells by supporting BCR and cytokine signaling and their downstream effectors (13). Consistent with this notion, NOTCH1 activity has been previously shown to synergize with BCR signaling to enhance B-cell activation, suggesting that NOTCH1 signaling might amplify BCR-mediated positive selection events (108).

All these abnormal proliferative signals along with epigenetic changes such as aberrant DNA methylation (109, 110) might cause genomic instability in CLL cells and render DNA more prone to genetic lesions, which represent other important leukemogenic events (25, 111). Genetic lesions include both chromosomal abnormalities such as deletions of chromosomes 13q, 11q, and 17p, trisomy 12, and gene mutations. The most frequent mutations in CLL were found within *TP53, NOTCH1, SF3B1, MYD88, ATM*, and *BIRC3* genes involved in the regulation of key biologically relevant pathways such as DNA repair and cell-cycle control, NOTCH1 signaling, inflammatory pathways, Wnt signaling, and RNA splicing and processing (112). As outlined in this review, *NOTCH1* was the most commonly mutated among these genes, with a high frequency in aggressive subsets of *IGHV*-unmutated CLL expressing particular stereotyped BCRs (113–115). These findings suggest the potential role of specific stereotyped BCR patterns in promoting the occurrence or selection of *NOTCH1* mutations influencing the outcome of CLL. Interestingly, whereas *TP53* mutations were found mainly as subclonal events that expanded over time favoring CLL progression and therapy resistance (33, 92, 116), *NOTCH1* mutations were either clonal, representing early events in CLL development, or sublconal, indicative of an occurrence at late steps in CLL development and of a selection during disease progression (33, 112). In a temporal study investigating the clonal architecture in CLL, the acquisition of *NOTCH1* mutations was classified as late event being preceded by trisomy 12, del(17p), and del(11q) initial hits (117). Our data indicated that subclonal *NOTCH1* mutations did not appear as temporary events as they identified high-risk patients (66). Rasi et al. reported that subclones harboring *NOTCH1* mutations displayed sensitivity to chemotherapy and did not gain a competitive advantage over the wild-type clones (93). Consistent with these studies, Ouillette et al. presented data supporting that *NOTCH1* mutations did not confer strong selective growth advantages on CLL cells and were not preferentially associated with CLL relapse or CLL clonal dominance (118). Altogether, these findings indicate the need for further investigations to support the role of NOTCH1 alterations in the transformation process and clonal evolution of CLL deriving from a mature B cell.

### NOTCH1 Alterations in CLL Hematopoietic Stem/Progenitor Cells: A New Theory on CLL Cellular Origin

Recent findings revolutionized the concept that CLL was a disease arising from a mature B cell, indicating the involvement of a hematopoietic stem cell (HSC) in the transformation process. The first report which challenged the theories on the origin and pathogenesis of CLL demonstrated that HSCs purified from the bone marrow of CLL patients and transplanted into xenograft models had a propensity to generate a CLL-like MBL (119), which is considered the pre-leukemic state of CLL (120, 121). In transplanted mice, CLL-HSCs gave rise to higher number of pro-B cells compared with healthy HSCs, suggesting that the CLL-HSCs were skewed toward a B-cell lineage commitment. In addition, the lymphoid expansions occurring in mice were clonally unrelated to the original CLL patient indicating a *de novo* generation that was probably initiated by genetic abnormalities already acquired at the long-term self-renewing CLL-HSC level (119).

The involvement of NOTCH1 alterations in CLL initiation was shown for the first time by a genomic analysis that revealed the presence of *NOTCH1* mutations in early hematopoietic progenitor cells of CLL patients with *NOTCH1* mutation (122). HSCs and the early hematopoietic progenitors of CLL patients also carry mutations in *SF3B1, BRAF, EGR2, MYD88*, and *NFKBIE* genes that are known to be mutated not only in CLL but also in other hematological malignancies (123–125). The evidence that *NOTCH1* and the above indicated mutations occurred in a progenitor able to undergo both lymphoid and myeloid differentiation also suggested that they might contribute to a pre-leukemic HSC stage, similar to genetic alterations observed in myeloid neoplasms (126, 127). The mechanism through which pre-leukemic HSCs contribute to CLL development is still unknown. We speculate that *NOTCH1* mutations might lead to the development of pre-leukemic mature B cells and increase the possibilities of acquiring further oncogenic events within a specific microenvironmental context or a transformation-permissive cellular background. Recent NGS studies demonstrated that *NOTCH1* mutations appeared at the CD34+/CD19− progenitor and CD34+/CD19+ pro-B cell level (128). This observation suggests that *NOTCH1* mutations may contribute to the expansion of early CLL hematopoietic progenitors representing one of the factors associated with the larger number of pro-B cells detected in the bone marrow of CLL patients compared with healthy donors (119).

Interestingly, we recently showed that CD34+/CD38− cells from CLL patients expressed NOTCH1 receptor and displayed higher levels of the active NOTCH1-ICD than healthy donors, independent of the *NOTCH1* mutational status (129). These data suggested that NOTCH1 activation started in the CLL stem cell compartment anticipating the occurrence of the mutation. Further studies are needed to understand the mechanisms underlying NOTCH1 activation in CLL-HSCs. A role may be played by various NOTCH1 ligands expressed in bone marrow mesenchymal stromal cells of CLL patients (130). However, whether their expression levels in these cells are higher than those in the normal bone marrow cells, and the specific NOTCH1 ligand that is involved in activating NOTCH1 signaling in CLL-HSCs, remain to be defined.

The discovery of NOTCH1 activation in CLL-HSCs and the presence of deregulated pre-BCR signaling driven by *BRAF* and *EGR2* mutations in early CLL hematopoietic progenitors (122) led us to hypothesize that NOTCH1 alterations might cooperate with aberrant pre-BCR signals to favor the occurrence of the premalignant mature B clones. Therefore, the cooperation between NOTCH1 and BCR might be important even for early CLL leukemogenesis.

**Figure 6:**
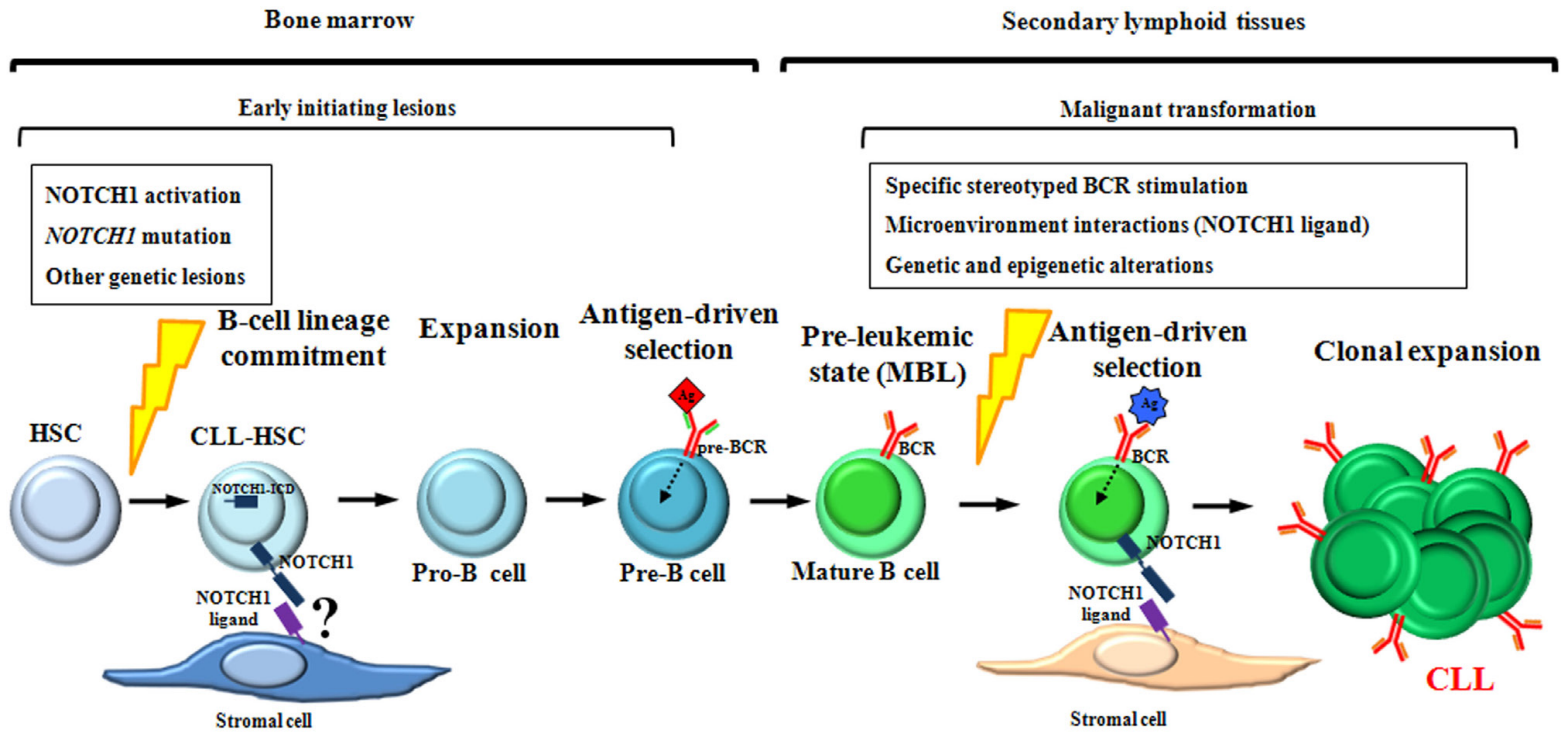
Schematic representation of NOTCH1 involvement during chronic lymphocytic leukemia (CLL) development. NOTCH1 activation and mutation are early oncogenic events acquired within hematopoietic stem cells (HSCs) and maintained in their hematopoietic progeny. As suggested by xenograft models, CLL-HSCs are skewed toward B-cell differentiation giving rise to premalignant B cells that resemble human MBL, the pre-leukemic state of CLL. We hypothesize that NOTCH1 alterations, together with other oncogenic events, contribute to the generation of pre-leukemic mature B cells by favoring the expansion of pro-B cells and by cooperating with pre-BCR signaling during the antigen-driven selection of pre-B cells. In lymphoid tissues, antigenic stimulation of specific stereotyped BCRs, in combination with NOTCH1-related microenvironment interactions and additional genetic lesions, might have a role in the transformation process of pre-leukemic mature B cells into CLL.

Although the sequence of the events underlying CLL development is far from being clear, all the findings above suggest a model for CLL development in which NOTCH1 alterations are involved in crucial steps of both HSCs differentiation and mature B-cells activation (Figure 6).

All these evidences may have therapeutic implications, suggesting that an anti-NOTCH1 treatment might be able to kill not only mature CLL cells but also their corresponding leukemia stem cells, favoring disease eradication for a definitive cure.

### Upstream Signaling Pathways Promoting NOTCH1 Activation in CLL

Little is known regarding the upstream pathways responsible for the deregulated NOTCH1 activation in CLL. Several lines of evidence suggest that NOTCH1 activation in CLL cells is under the control of microenvironmental conditions through ligand-dependent mechanisms. Indeed, the NOTCH1 ligands expressed on stromal cells in the bone marrow (130) or in lymph nodes increased NOTCH1 activity and mediated CLL survival regardless of *NOTCH1* mutational status (107). The importance of microenvironmental signals in inducing NOTCH1 cascade is further documented by the evidence that CLL cells in the lymph nodes frequently express NOTCH1-ICD independent of *NOTCH1* mutation (69, 131), especially within the proliferation centers, which represent the key microanatomical sites where CLL cells interact with accessory cells and acquire chemoresistance. Microenvironmental interactions are critical for NOTCH1 activation not only in *NOTCH1* wild-type but also in *NOTCH1-*mutated patients (107).

It has been shown in several malignancies that there is a complex crosstalk between NOTCH1 and the transcription factor nuclear factor-kappa B (NF-κB) (132), another key pathway involved in cancer (133) and in CLL pathogenesis (134). Furthermore, NF-κB can indirectly trigger the NOTCH1 signaling pathway by inducing the expression of the NOTCH1 ligand JAGGED1 during B cell activation (135). In a previous study aimed to explore the mechanisms underlying NOTCH1 activation in CLL cells devoid of *NOTCH1* mutations, we excluded the possibility that genetic lesions of NF-κB regulators or *JAGGED1* and *JAGGED2* genes were involved in NOTCH1 activation in these leukemic cells (136).

Secchiero et al. suggested a potential role of the tumor suppressor protein p53 in the induction of NOTCH1 pathway in CLL (137). They showed that the anti-proliferative and pro-apoptotic agent nutlin-3 resulted in a p53-dependent increase of NOTCH1 mRNA and protein levels in CLL cells. These results, along with the findings that γ-secretase inhibitors (GSIs) increased the cytotoxicity of nutlin-3, suggested that the p53-mediated expression of NOTCH1 initiated an antiapoptotic feedback mechanism limiting the cytotoxic actions of nutlin-3. The p53 dependence of nutlin-induced NOTCH1 expression was confirmed by the observation that the increase in NOTCH1 occurred in *TP53* wild-type myeloid and lymphoid leukemic cell lines but not in cell lines lacking a functional *TP53* gene. Furthermore, whereas the silencing of p53 expression abrogated the induction of NOTCH1 by nutlin-3, the silencing of NOTCH1 enhanced the cytotoxic effect of nutlin-3, highlighting that NOTCH1 was an antiapoptotic target of p53 in both lymphoid and myeloid leukemia cells (137).

There is evidence that various recurrent gene mutations lead to a dysregulated NOTCH1 activation in CLL. In a transcriptomic analysis aimed to characterize the functional impact of *SF3B1* mutations on CLL, Wang et al. identified NOTCH1 signaling as one of the pathways affected by this mutation (138). Specifically, they demonstrated that *SF3B1* mutations increased NOTCH1 signaling through altered splicing of *DVL2*, a core canonical Wnt pathway member and negative regulator of NOTCH1 activation. Mutations in the *FBXW7* gene can also deregulate the NOTCH1 signaling pathway in CLL. *FBXW7* encodes an E3 ubiquitin ligase that regulates the stability of NOTCH1-ICD by targeting it for ubiquitination and degradation (139). Inactivating mutations of *FBXW7* are observed in 2.5% of CLL cases (36), suggesting a role of genetic aberrations in the NOTCH1-ICD degradation machinery in CLL pathogenesis (32, 140). It has been recently reported that even *MED12* mutations contributed to activate NOTCH1 signaling in CLL (38). MED12, MED13, CDK8, and cyclin C form a four-subunit kinase module that is associated with a 26-subunit mediator core complex, which regulates many transcriptional programs important for development and/or tumorigenesis (141). CDK8 represses NOTCH1 signaling-driven transcription by phosphorylating the PEST domain of NOTCH1-ICD, an event required for its ubiquitination by the E3 ligase FBXW7 and subsequent degradation (142). It has been proposed that the increased levels of NOTCH1-ICD detected in CLL cells in the context of *MED12* mutations are mediated by an aberrant CDK8 kinase activity (38). Inactivating mutations of the *SPEN* gene, which were detected in approximately 1% of CLL cases, also contributed to increase NOTCH1 activation (20, 32). SPEN is a co-repressor of RBPJ, the nuclear effector of the NOTCH1 pathway, and a putative negative regulator of NOTCH1 signaling (143).

These latter studies provided important information about the genetic background in which NOTCH1 activation occurred in CLL, but the current knowledge about the regulation of NOTCH1 signaling in CLL does not explain the mechanisms underlying the constitutive expression and activation of NOTCH1 in peripheral blood CLL cells lacking *NOTCH1* mutation. We hypothesize that one of these mechanisms may involve NOTCH1-ligand interactions between CLL cells themselves, given that they also constitutively express the ligands JAGGED1 and JAGGED2 (19, 144). However, these interactions may also not contribute to NOTCH1 activation, because, as reported in other cell types, when NOTCH1-ligand interactions occur within the same cell (*cis*-interactions), they may lead to suppression rather than activation of NOTCH1 signaling (145, 146). Another possible mechanism underlying the constitutive NOTCH1 activation in circulating CLL cells may be ligand independent through a disrupted endosomal trafficking and an aberrant regulation of NOTCH1 receptor during its recycling, ubiquitination, and degradation (147–149). Finally, we cannot exclude the possibility that other pathways relevant for CLL pathogenesis, including the NF-κB signaling (150) and those triggered by stimulation of BCR, cytokine/chemokine receptors, or CD40 molecule, are involved in activating NOTCH1 in CLL. In this context, we demonstrated that interleukin-4, a T cell-derived cytokine involved in CLL pathogenesis and known inducer of CLL cell survival (151), potentiated NOTCH1 expression and activation in promoting its prosurvival effect (152).

### Pathogenic Role of *NOTCH1* Stabilizing Mutations in CLL

The loss of the PEST domain by *NOTCH1* mutations has been predicted to result in NOTCH1-ICD impaired degradation with its consequent stabilization and increased NOTCH1 signaling (29). One of the first demonstrations of the functional impact of *NOTCH1* mutations in CLL was provided by Arruga et al., who revealed the presence of the truncated NOTCH1-ICD protein encoded by the mutant *NOTCH1* allele in CLL cells. Compared with *NOTCH1*-wild-type cases, *NOTCH1*-mutated CLL cells displayed a more intense activation of the NOTCH1 pathway that conferred a marked resistance to drug-induced apoptosis (107). However, although it is well documented that *NOTCH1* mutation stabilizes NOTCH1 signaling in CLL by increasing the stability of the truncated NOTCH1-ICD (107), the molecular mechanisms underlying this effect are poorly defined. In this context, we demonstrated that, in *NOTCH1*-mutated CLL, the loss of the PEST domain altered the phosphorylation status of mutated NOTCH1 protein and generated a phosphorylated NOTCH1-ICD form that accumulated in the nucleus, leading to increased NOTCH1 signaling and prolonged CLL cell survival (153). It has been previously shown in other cell types that, whereas phosphorylation of PEST domain targeted NOTCH1-ICD for proteasomal degradation, attenuating its signaling (142), phosphorylation in regions upstream of the PEST domain could increase NOTCH1 signaling (154) and even mediate NOTCH1-dependent oncogenesis (155). We also demonstrated that NOTCH1-ICD phosphorylation is reversed by Idelalisib (153), a selective inhibitor of PI3Kδ, currently used for CLL therapy (83, 87, 156). These data suggest a possible involvement of the PI3Kδ oncogenic pathway in the phosphorylation of mutated NOTCH1-ICD, and, more important, that this event may represent a new potential therapeutic target in *NOTCH1*-mutated CLL.

### Downstream Effects of NOTCH1 Alterations in CLL

#### NOTCH1-Dependent Transcriptional Program

**Figure 7:**
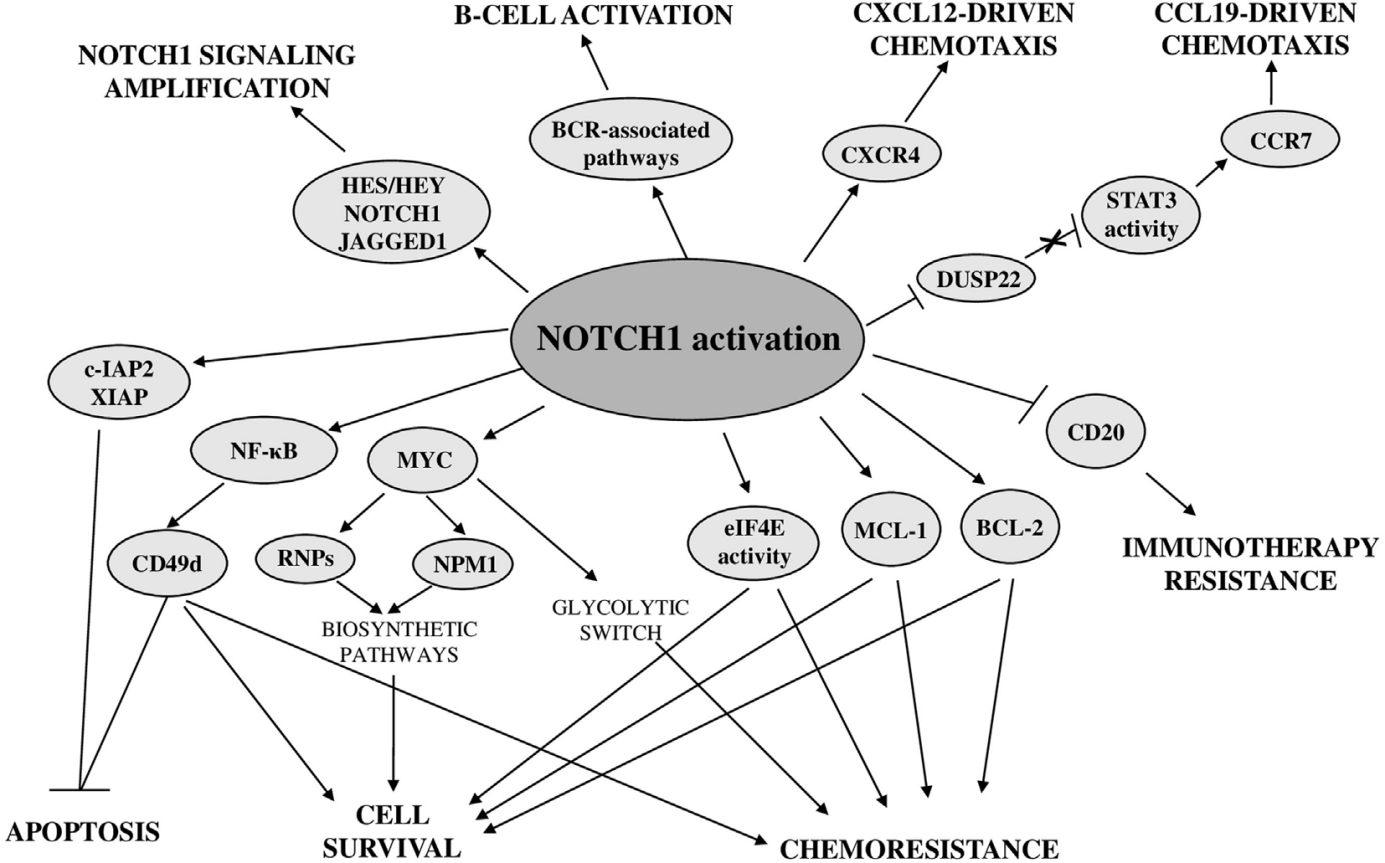
Biological consequences of deregulated NOTCH1 in chronic lymphocytic leukemia.

To shed light on the NOTCH1-controlled biological functions in CLL, a recent study investigated the NOTCH1-dependent CLL transcriptional signature. This comprehensive analysis revealed that NOTCH1 activation, independent of mutational status, leads to upregulation of a high number of transcripts, including known NOTCH1 targets, such as *HES/HEY* family members, *NRARP* and *DTX1*, antiapoptotic and cytokine-chemokine genes, as well as genes involved in immune and signaling pathways relevant for the development and activation of B cells (13) (Figure 7). Among these latter, there are BCR-associated pathway genes, including upstream pathway members (e.g., LYN, SYK, BLK, BLNK, and CR2), and downstream effectors, such as MAPK and NF-κB cascade members. Notably, NOTCH1 signaling also induces its own transcript and that of its ligand JAGGED1, that is known to be expressed in CLL cells (19), suggesting a positive feed-forward loop in NOTCH1 activation.

#### Effects on Antiapoptotic Pathways

Recent advances demonstrated a close relationship between oncogenic NOTCH1 signaling and the control of cell survival, proliferation, homing, and chemoresistance (Figure 7). Among antiapoptotic effectors downstream of NOTCH1 signaling, there is the transcription factor NF-κB whose overexpression and increased activity play a pivotal role in CLL pathogenesis by promoting tumor cell proliferation and survival (157–159). We provided the first evidence that in CLL cells, NOTCH1 signaling increased the activity of NF-κB and the expression of cellular inhibitor of apoptosis protein 2 (c-IAP2) and X-linked inhibitor of apoptosis protein(XIAP) (19). A role of NOTCH1 in activating NF-κB has been also demonstrated in recent studies performed in CLL cells with *NOTCH1* mutation (160). In addition, a NOTCH1-dependent activation of NF-κB has been shown to drive the expression of CD49d, a well-known key regulator of microenvironmental interactions and negative prognosticator in CLL (59). The effect of NOTCH1/NF-κB axis on CD49d expression is stronger in *NOTCH1*-mutated CLL cells, but it is not restricted to this CLL subset. The higher levels of CD49d expression and NF-κB activation in *NOTCH1*-mutated compared with *NOTCH1*-wild-type CLL are likely due to the higher NOTCH1 activation induced by mutations (7, 29, 107). Another important mechanism by which constitutive NOTCH1 signaling promotes CLL cell survival is by sustaining the expression of the antiapoptotic myeloid cell leukemia 1 (MCL-1) protein and the activity of the translational regulator eukaryotic translation initiation factor 4E (eIF4E) (152), which are both implicated in CLL pathogenesis (161, 162). NOTCH1 signaling does not regulate MCL-1 expression at the transcriptional level but by preventing its proteasomal degradation, indicating that this effect may be mediated by a non-canonical NOTCH1-ICD-activated signaling rather than by NOTCH1-ICD transcriptional activity (152).

Another antiapoptotic target of NOTCH1 signaling in CLL is BCL-2 (13), a factor with a well-established role in CLL pathogenesis (85). The increased NOTCH1 activation, induced in CLL cells cultured with bone marrow mesenchymal cells, confers chemoresistance by upregulating BCL-2 expression (130).

#### Effects on CLL Therapy Resistance

*NOTCH1* mutations are enriched among chemorefractory CLL patients indicating a potential relationship between deregulated *NOTCH1* and response to treatment. *In vitro*, NOTCH1 activation promotes CLL cell survival by sustaining the expression of MCL-1 and the activity of eIF4E, proteins that contribute to chemotherapy resistance (152). In addition, CLL cells from patients with mutated *NOTCH1* show a marked resistance to *in vitro* fludarabine-induced apoptosis, which is abrogated in the presence of NOTCH1 inhibitors (107, 163). All these findings highlight the importance of targeting NOTCH1 signaling for CLL treatment, especially in combination with agents, such as fludarabine, whose poor efficacy is mainly due to the elevated MCL-1 expression and eIF4E activity detected in these leukemic cells (162, 164).

The presence of *NOTCH1* mutations has been also associated with a relative resistance to anti-CD20 immunotherapy in a prospective clinical study comparing the effectiveness of the FC regimen versus the FC plus rituximab regimen (12). These clinical data are sustained by biological evidences that *NOTCH1*-mutated CLL cells are characterized by lower CD20 expression and lower lysis induced by anti-CD20 exposure *in vitro* (65). These studies showed that accumulation of mutated NOTCH1-ICD in the nucleus was responsible for a dysregulation of histone deacetylase (HDAC)-mediated epigenetic repression of CD20 expression, by altering the balance of the two functions of RBP-Jk as transcriptional activator when complexed with NOTCH1-ICD, or transcriptional repressor when complexed with HDACs. Specifically, in *NOTCH1*-mutated CLL cells, RBP-Jk was less complexed with HDACs which so were more bound to the CD20 promoter, resulting in epigenetic silencing of gene expression.

#### Effects on Pathways Regulating CLL Homing and Cell Growth

A NOTCH1-ICD-dependent epigenetic modulation of gene expression also affects other targets by influencing signaling pathways regulating growth and homing of CLL cells. It has been demonstrated that NOTCH1 signaling reduced the expression of the tumor suppressor gene *DUSP22* through a methylation-dependent mechanism. *DUSP22* downregulation led to constitutive activation of signal transducer and activator of transcription 3 (STAT3) signaling which increased the expression of CCR7 promoting CCL19-driven chemotaxis (165).

Analysis of NOTCH1-dependent transcriptional signature showed that even *CXCR4* is a direct target of NOTCH signaling in CLL (13). This gene encodes a chemokine receptor, highly expressed in CLL cells, relevant for their chemotaxis toward microenvironmental cells producing the CXCL12 ligand (166). The CXCL12/CXCR4 axis is crucial for the dissemination of CLL cells to lymphoid organs and has been shown to be associated with poor prognosis (167).

Another target gene of NOTCH1 transcriptional activity is *CCND3* (13) that encodes a cyclin involved in G1/G2 transition, suggesting a role of NOTCH1 in CLL cell proliferation. Gene expression profiling studies demonstrated that NOTCH1 activation might confer cell growth and/or proliferation advantages to CLL cells even by upregulating genes related to ribosome biogenesis such as nucleophosmin 1 (*NPM1*) and ribosomal proteins (*RNPs*) (70). These effects were mainly associated with *NOTCH1* mutation but were also observed in *NOTCH1*-wild-type CLL cells cocultured with JAGGED1-expressing stromal cells. Bioinformatics analyses and *in vitro* activation/inhibition of NOTCH1 signaling suggested a role of MYC as a mediator of NOTCH1 effects on NPM1 and RNP expression in CLL cells. Chromatin immunoprecipitation experiments performed on NOTCH1-ICD transfected CLL-like cells showed the direct binding of NOTCH1 to the *MYC* promoter and transfection with MYC-specific small interfering RNA reduced NPM1 expression, confirming that MYC was a transcriptional target of the NOTCH1 activation complex in CLL. Furthermore, the evidence that modulation of NOTCH1 signaling directly influences MYC transcript levels corroborates the hypothesis that a mutation-dependent increase of NOTCH1 activation may be responsible for a higher MYC-dependent transcription of *NPM1* and *RNPs* (168). Activation of a NOTCH1–c-Myc axis is also involved in a glycolytic switch induced in CLL cells by stromal cells, contributing to stroma-mediated chemoresistance (169). Targeting glucose metabolism may represent a new therapeutic approach for CLL with deregulated NOTCH1 to overcome stromal cell-mediated drug resistance in this disease.

## Future Perspective: Therapeutic Targeting of NOTCH1 in CLL

Despite increasing insight into its tumor biology, CLL remains an incurable disease. Currently, immunochemotherapy is the standard of care for treatment-naïve patients (170), as it significantly improves clinical outcome. However, this approach is associated with adverse events and yields poor results in those patients with high-risk features. BCR and BCL-2 inhibitors are revolutionizing the treatment landscape of this disease (79, 83, 85), indicating that a molecularly targeted therapy can lead to high-rate improvement of outcome in CLL. However, several issues limit the advances of new CLL inhibitors, including the inability to eradicate the tumor and resistance/progression, suggesting the need for alternative treatment approaches.

The growing evidence for a critical role of the NOTCH1 pathway in CLL makes this cancer gene a target to design a custom-made treatment for this blood disease. The most promising opportunity derived from the discovery of NOTCH1-deregulated signaling and mutations in CLL is the development of anti-NOTCH1-targeted therapies. Notably, a broad number of patients may benefit of an anti-NOTCH1 therapy, given the importance of enhanced NOTCH1 signaling in CLL, even without carrying a *NOTCH1* mutation (13).

The NOTCH1 pathway is highly regulated at multiple steps; thus, a number of genetic and pharmacological strategies are available to block or silence this signaling network. Currently, GSIs are the most extensively explored anti-NOTCH1 molecules in different cancers. Treatment with GSIs induces apoptosis by inhibiting the proteolytic system responsible for the activation of NOTCH1 receptors. A considerable number of early phase trials demonstrated the anti-cancer efficacy of GSIs in solid tumors. In CLL, the use of GSI-I exhibited cytotoxicity on leukemic cells coupled with downregulation of NOTCH1 activity *in vitro* (171). Similarly, the combination of the clinically relevant GSI PF-03084014 and fludarabine demonstrated antitumor effects in primary *NOTCH1*-mutated CLL cells (163).

Concerns about off-target toxicity (172, 173) delayed the clinical translation of GSI-based therapy, including CLL treatment, and suggested the need of more selective antagonists. Several antibodies blocking the activity of individual NOTCH1 receptors have been developed for the treatment of solid tumors, demonstrating limited toxicity compared with GSIs. A humanized antibody targeting NOTCH1 (OMP-52M51) did not affect intestinal goblet cells’ differentiation in pre-clinical studies (174, 175) and entered phase I trials in solid tumors and relapsed/refractory lymphoid malignancies (NCT01778439, NCT01703572). Although promising, translation of these results into novel therapeutic approaches for CLL with aberrant NOTCH1 activation will require further investigation, given a limited value shown in the contest of T-ALL (175).

Recently, the search for alternative approaches to a GSI-based NOTCH1 inhibition led to the identification of new NOTCH1 modulators in T-ALL (176). In CLL, we use a similar approach to demonstrate the capacity of the small molecule bepridil to preferentially target NOTCH1 over NOTCH2 and induce apoptosis *in vitro* and *in vivo* (177). However, bepridil is a known calcium channel blocker with potential toxic effects in non-diseased cells that may limit its repositioning in CLL targeted therapy.

On-target delivery remains the principal obstacle in developing anti-NOTCH1 drug approaches. As for most cancer mutations and deregulated pathways, NOTCH1 is not an obvious drug target, given its role in different biological processes and cell types (15, 178). This represents a potential limit for an anti-NOTCH1 therapy, as ubiquitous targeting of NOTCH1 in non-leukemic cells may trigger toxic effects. In CLL, nanoparticle-based drug delivery platforms have emerged as suitable vehicles for specific targeting cytotoxic drugs against the CD20 expressed on neoplastic B cells (179). The use of nanotechnology represents a potential approach (180) to help improving the selectivity, effectiveness, and safety of molecules that inhibit NOTCH1.

Finally, the efficacy of NOTCH1 targeting may be also reduced by intraclonal heterogeneity of the CLL clones. Indeed, subclones with genetic lesions other than *NOTCH1* mutations frequently coexist in CLL (117). This limit implies the need to test combinations of anti-NOTCH1 molecules with drugs targeting different components of the molecular network in CLL cells. Preliminary evidence shows that GSIs enhance the antileukemic activity of the BTK inhibitor Ibrutinib in CLL cells (181). We believe that the future of CLL treatment lies in the association of small molecule inhibitors targeted at the BCR pathway and the antiapoptotic BCL-2 protein. Upcoming research efforts will need to investigate the value and potential integration of NOTCH1 targeted agents into this CLL treatment algorithm.

## Author Contributions

ER and PS organized the plan and structure of the manuscript, and all the authors contributed to the redaction.

## Conflict of Interest Statement

The authors declare that the research was conducted in the absence of any commercial or financial relationships that could be construed as a potential conflict of interest.
